# The sociodemographic characteristics and dietary and blood plasma fatty acid profiles of elderly Saudi women with Alzheimer disease

**DOI:** 10.1186/s12944-019-1029-0

**Published:** 2019-03-30

**Authors:** Samar Rashed Alsumari, Doha Mustafa AlNouri, Mervat Mohamed Ali El-Sayed, Mohamed Fekry Serag El-Din, Shaista Arzoo

**Affiliations:** 1 Department of Food and Nutrition Sciences, College of Food and Agriculture Sciences, King Saud University, Riyadh-11495, P.O. 11495, Riyadh, Saudi Arabia; 2 Department of Nutrition and Food Science, Faculty of Home Economics, Menoufia University, Shebin El Kom, Egypt

**Keywords:** Alzheimer, Fatty acids, Brain, Plasma, Correlation, Diet

## Abstract

**Background:**

Alzheimer’s disease (AD) is a progressive neurodegenerative disease, and due to various physiological and psychological factors the patients are at risk of nutritional insufficiencies. The purpose of this study was to assess the dietary fatty acid intake and its effect on plasma fatty acids in elderly Saudi women and to compare the differences in their food and plasma fatty acid profile on the basis of their residence.

**Methods:**

A total of 76 elderly women (50–100 years) were recruited through a random sampling method. A structured proforma was designed to gather information related to their age, income, dietary habits, and presence of any disease and awareness of AD. A 24-h dietary recall method for 3 days and food frequency questionnaire, concentrating on fish consumption and consumption of foods rich in ω-3 fatty acids, which was planned by dietitians, was used for dietary assessment. The gathered data were then analyzed using food processor software. The blood samples were collected to determine plasma fatty acids.

**Results:**

The mean age of women diagnosed with AD was more than 75 years, and the prevalence of illiteracy was higher among AD subjects. As compared to the AD group, the concentration of LA and total ω-6 was significantly (*p* ≤ 0.05) higher in the control group from both recruitment sites [National Guard Health Affairs, King Abdulaziz Medical City, Riyadh (NGH) and Social Welfare Homes for the Elderly (SWH)]. Similarly, the concentrations of EPA, DHA, and ω-3 were also slightly higher in the control group at both sites, but the difference between the control and AD subjects was only significant (*p* ≤ 0.05) in subjects from NGH. We found no significant difference in the ω-6/ ω-3 ratio between groups. Also, no significant difference was found in the mean level of the plasma fatty acid when comparing the control and AD groups. The concentration of DHA in controls only and AA, EPA and ω-6 in both control and AD were significant (although weakly) correlated with their respective dietary intakes. No correlations were found between the intake of 18 C precursors (LA and ALA) and plasma levels of their long chain derivatives (AA, EPA, and DHA). Education, income, overall health status and the concentration of various fatty acids from food was higher and better in subjects from SWH than NGH. The lower plasma level indicates lower impaired systemic availability of several nutrients.

**Conclusion:**

We found that dietary intervention might play a role in the prevention of AD.

## Background

Alzheimer disease’s (AD) is a progressive multifarious neurodegenerative disorder, characterized by intracellular neurofibrillary tangles and extracellular amyloidal protein deposits contributing to senile plaques [1, 2]. The threat of developing AD is rising exponentially with age and is the main cause of dementia and the most common neurodegenerative disease in the elderly [3]. Oxidative stress and inflammation are the underlying mechanisms of AD pathology [4]. The number of AD suffers is expected to reach 106.8 million worldwide by the year 2050. Therefore, it is a growing public health concern and a major socioeconomic burden [5]. The most common symptoms shown by AD patients include memory loss, along with impairment in reasoning, visual perception, language ability or attention [6].

The human brain is primarily lipid with 22% of the cerebral cortex and 24% of white matter comprising of phospholipids. Although the brain proteins are fixed by the genetic code, the fatty acid composition of the brain phospholipids can be modified. Several mechanisms have been proposed for the defensive role of omega 3 fatty acid in dementia. First, sufficient omega-3 fatty acid might help sustain integrity and neuronal function. Second, DHA amends the expression of genes that regulate a variety of biological functions or cognitive health, including neurogenesis and neuronal function. Third, the oxidative products of PUFAs act as key cellular mediators of inflammation, oxidative stress, allergy and immunity, vascular responses, and thrombosis [7]. Studies have revealed that dietary deficiency of n-3 fatty acid or linoleic acid together with linolenic acid leads to decreased brain phospholipid arachidonic acid and DHA, with concomitant rises in brain n-9 and n-7 MUFAs and PUFAs [8–10]. Although various studies have been conducted to examine the relationships between dietary fat intake and the development of AD, results have been inconsistent as; some have reported strong evidence of association while others have not [11–13]. The main purpose of this study was to assess the dietary fatty acid intake and its effect on plasma fatty acids in elderly Saudi women with and without AD, and to compare the differences in their food and plasma fatty acid profile on the basis of their shelter/residence.

## Materials and methods

### Research design

A descriptive cross-sectional study approach was used to assess the dietary fatty acid intake and its effect on plasma fatty acids in elderly women.

### Sample and sampling technique

**Fig. 1:**
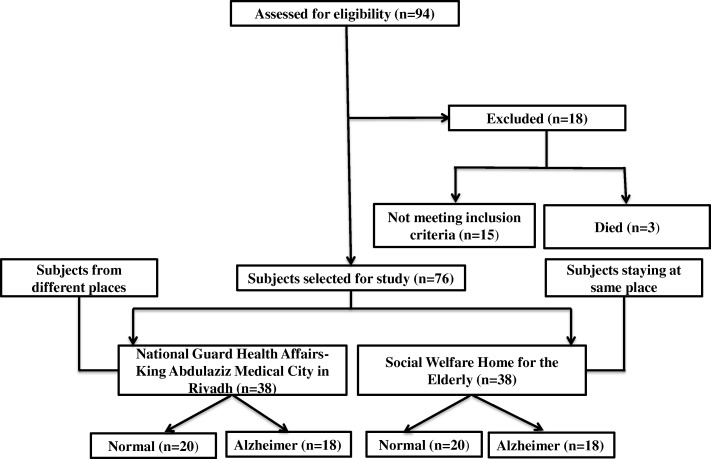
Flow of participants through the study

A total of 76 elderly women (50–100-years-old) were recruited through a random sampling method (Fig. 1). A hospital [National Guard Health Affairs, King Abdulaziz Medical City, Riyadh (NGH)] and an old age home Social Welfare Homes for the Elderly (SWH)] were randomly selected, and a survey was made to identify the women of the required age. Then, through a lottery method, the desired sample of women was chosen by a person aware of the study aims [14].

### Inclusion and exclusion criteria

Women between 50 and 100-years-old, non smokers were included in the study. All of the women more than 50- and less than 100-years-old, smokers, and women taking any supplementation were excluded from this study.

### Demographic characteristics

A structured proforma was designed and completed with subjects consent while interviewing them to get their demographic information. Their names were not required on the questionnaire and subjects were assured that the information given was entirely for scientific purposes and would be kept confidential. A set of multiple-choice questions related to their age, income, the presence of any disease, awareness of AD, and consumption of certain omega 3, 6 and 9 fatty acid [approved by a panel of experts (in Arabic)] were used to record the data.

### Anthropometric measurements

Height and weight (mentioned on the record book of subjects) were used to calculate BMI using the formula: BMI = weight (kg)/height^2^ (m)^2^. The women were classified as underweight if their BMI was < 18.5, normal if it was between 18.5 and 24.9, overweight if it was 25–29.9, and obese if their BMI was greater than 30 [15].

### Dietary assessment

A 24-h dietary recall method for 3 days and food frequency questionnaire, concentrating on fish consumption and consumption of foods rich in ω-3 fatty acids, as planned by dietitians, was used for dietary assessment, which was then analyzed using food processor software. Subjects were interviewed by the nutritionist to report all food and beverages they had consumed during 24 h (for 3 days). Then, each item was described as much as possible. The subjects were instructed to report everything eaten or drunk for the past 24 h. The questionnaire included details regarding the exact portion of the food consumed (serving size), type of food (such as low fat, full fat, skimmed, sugar free, fortified), the method of preparation (boiled, deep-fried, baked, grilled), the parts consumed (whole, half, quarter), and the brand name (commercial, ready to eat) [16–18].

### Collection of blood samples

The blood samples were collected after 12-h fasting in lithium heparin tubes and centrifuged at 2500 rpm for 5 min in a megafuge. After centrifugation, the supernatant was separated and stored at − 80 °C for further analysis.

### Methylation and identification of fatty acids (FA)

Samples were prepared and analyzed according to the method described by Folch et al. [19]. Identification of the individual FA methyl esters was achieved using reference standards. Standards of fatty acids were purchased from Sigma-Aldrich. The FA composition was analyzed using a gas chromatograph (Agilent, GC Mass 6890 model, Schimadza Co. Japan). The gas chromatographic parameters were as follows: Column, fused silica capillary (omegawax, HP-5MS, 30 m × 0.25 mm × 0.25 mm); Carrier gas, Helium, 1 ml/min; Detector, M/S –Agilent, 250 °C; Programming temperature, 70–280 °C, after 2 min increased at 20 °C/2 min to the maximum 280 °C.

### Statistical analysis

Data were analyzed using the SPSS statistical software package and expressed as the mean ± standard deviation. The differences among the dietary treatment groups were analyzed by one-way ANOVA at a significance level of *p* ≤ 0.05. If significant differences were found, a post-hoc analysis using Duncan’s multiple range tests was performed.

## Results

### Sociodemographic data and dietary habits of studied subjects

Table 1 depicts the socio-demographic and health characteristics and dietary habit of the studied samples. The mean age of women diagnosed with AD was more than 75 years, and the mean age of women from the control group was less than 75 years. The BMI of subjects suffering from AD was less than in the control group. Similarly, the prevalence of illiteracy was higher among women suffering from AD. None of the studied subjects had a degree higher than intermediate. Financially, subjects from the control groups were more stable than the AD patients. For all groups, the most prevalent systemic diseases were diabetes and hypertension, followed by heart disease. Almost 39% of the AD subjects from NGH had both diabetes and blood pressure, and this proportion was 44.44% for the AD subjects from SWH, both of which are greater than the control group (20% from NGH and 40% from SWH). Although women from the control groups were aware of AD and ω-6 and ω-3, none of the AD patients had heard these words. Education level, income, and overall health status were higher and better in subjects from SWH.

**Table 1:** Socio-demographic and health characteristics and dietary habit of studied subjects

Variables	National Guard Health Affairs- King Abdulaziz Medical City, Riyadh (NGH)		Social Welfare Homes for the Elderly (SWH)	
	Control	Alzheimer	Control	Alzheimer
Socio-demographic Characteristics				
Age (mean ± SD)	69.40 ± 9.29	76.00 ± 13.05	74.65 ± 10.45	80.17 ± 8.33
BMI (mean ± SD)	30.37 ± 8.37	24.23 ± 8.49	29.15 ± 7.35	28.49 ± 5.06
*Education Level*				
Illiterate	70%	94.44%	65%	77.78%
Elementary	30%	5.56%	20%	16.67%
Intermediate	0%	0%	15%	5.56%
Higher	0%	0%	0%	0%
*Income*				
Less than 3000	95%	100%	25%	27.78%
3000–5000	5%	0%	65%	44.44%
More than 5000	0%	0%	10%	27.78%
Chronic non communicable and health conditions				
Diabetes	30%	22.2%	20%	27.78%
Diabetes + Blood pressure	20%	38.89%	40%	44.44%
Arthritis	10%	0%	0%	0%
Heart Disease	5%	16.67%	5%	11.11%
Any other	4%	5.56%	2%	0%
Do know what is Alzheimer	10%	0%	50%	0%
Do know what is ω-6 and ω-3	5%	0%	25%	0%
Dietary habit				
Fish				
Do not eat at all	0%	5.56%	15%	27.78%
Everyday	0%	0%	0%	0%
Per week	60%	33.33%	30%	22.22%
Per month	40%	61.11%	55%	50%
Egg/day	65%	45%	75%	25%
Meat/day	25%	22%	38%	32%
Chicken/day	40%	37%	57%	49%
Nut/day	0%	0%	0%	0%
Flaxseed /day	0%	0%	0%	0%
Sesame oil/day	0%	0%	0%	0%
Sunflower oil/ day	55.55%	44.50%	70.75%	29.25%
Olive oil/day	80.45%	19.55%	68.60%	31.4%
Corn oil/day	0%	0%	0%	0%
Supplementary food (fish oil)	0%	0%	0%	0%

It can be observed from Table 1 that 5.56% of the AD patients from NGH and 27.78% of AD patients from SWH never consume fish. The majority of the subjects, both from the control and AD groups prefer to consume fish monthly. Compared to the control group, the consumption of ω-6 and ω-3 rich food was less in the AD patients. None of the subjects consumed nuts, flaxseed, sesame oil, corn oil, or supplementary food (fish oil) daily, while the daily consumption rates of eggs, chicken, and red meat were higher in the control group. When control subjects from the two tested sites were compared, the consumptions of fish per week or per month, meat or chicken per day, and sunflower oil per day were found to be higher in subjects from SWH. When AD subjects from the two tested sites were compared, a greater percentage (27.78%) of AD patients from SWH were found not to consume fish at all, and the consumption of eggs was also lesser in subjects from SWH than those from NGH.

### Food and plasma fatty acid concentration

The fatty acid concentrations of various foods are shown in Table 2. Compared to the AD group, the concentrations of LA and total ω-6 were higher (*p* ≤ 0.05) in the control group (both sites, NGH and SWH). Similarly, the concentrations of EPA, DHA, and ω-3 were also slightly higher in the control group at both sites (NGH and SWH), but the difference between the control and AD subjects was only significant (*p* ≤ 0.05) in subjects from NGH. We detected no significant difference in the ω-6/ ω-3 ratio when comparing the control and AD groups at both sites (NGH and SWH). No significant differences (except EPA) were observed when the food fatty acid profiles of control subjects from both places were compared. Similarly, statistically insignificant (*p* ≥ 0.05) differences (except AA, EPA, and ω-3) were observed when the food fatty acid profiles of AD subjects from both sites were compared. Compared to the NGH, the concentrations of LA, AA, ALA, EPA, DHA, total ω-3, and total ω-6 were higher in AD patients recruited from SWH.

**Table 2:** Fatty acid profile of food in control and AD* patients

Fatty acid from Food (g/day)	National Guard Health Affairs- King Abdulaziz Medical City, Riyadh (NGH)		Social Welfare Homes for the Elderly (SWH)	
	Control	Alzheimer	Control	Alzheimer
LA (Linoleic acid; C18:2)	31.77 ± 2.902^Aa^	25.21 ± 2.221^Bb^	30.80 ± 3.0319^Aa^	26.34 ± 2.0249^Bb^
AA (Arachidonic acid; C20:4)	0.081 ± 0.006^Aab^	0.067 ± 0.0015^Bb^	0.077 ± 0.0022^Aab^	0.089 ± 0.0060^Aa^
ALA (α-linolenic acid; C18:3)	0.924 ± 0.0103^Aa^	0.816 ± 0.0240^Aa^	0.920 ± 0.0105^Aa^	0.844 ± 0.0216^Aa^
EPA (Eicosapentaenoic acid; C20:5)	0.050 ± 0.0051^Aab^	0.023 ± 0.0017^Bb^	0.071 ± 0.0075^Aa^	0.052 ± 0.0046^Aab^
DHA (Docosahexaenoic acid; C22:6)	0.012 ± 0.0005^Aa^	0.007 ± 0.0005^Ba^	0.013 ± 0.0006^Aa^	0.013 ± 0.0014^Aa^
Total ω-6	31.85 ± 2.908^Aa^	25.27 ± 6.23^Bb^	30.87 ± 3.0332^Aa^	26.43 ± 2.0265^Bb^
Total ω-3	0.986 ± 0.0117^Aa^	0.846 ± 0.0260^Bb^	1.002 ± 0.0126^Aa^	0.909 ± 0.0253^Aab^
ω-6/ ω-3	32.30 ± 2.279^Aa^	29.86 ± 2.552^Aa^	30.80 ± 3.0893^Aa^	29.075 ± 2.0848^Aa^

*AD-Alzheimer disease; Results are reported in mean ± standard deviation

^ab^Different superscripts denote a statistically significant difference between controls of two different places and between Alzheimer patients of two places

^AB^Different superscripts denote a statistically significant difference between controls and Alzheimers patients of each place individually

In this study, comparing control and AD groups, we detected no statistically significant differences were detected in the mean level of the plasma fatty acid (Table 3). Similarly, difference’s in the mean levels of plasma fatty (except EPA) was statistically insignificant (*p* ≥ 0.05) when comparing the NGH and SWH recruits, both control and AD.

**Table 3:** Fatty acid profile of plasma in control and AD* patients

Fatty acid from plasma (g/day)	National Guard Health Affairs- King Abdulaziz Medical City, Riyadh (NGH)		Social Welfare Homes for the Elderly (SWH)	
	Control	Alzheimer	Control	Alzheimer
LA (Linoleic acid; C18:2)	2.423 ± 0.1532^Aa^	1.554 ± 0.1500^Aa^	1.899 ± 1.001^Aa^	01.756 ± 0.1080^Aa^
AA (Arachidonic acid; C20:4)	0.019 ± 0.0007^Aa^	0.002 ± 0.018^Aa^	0.017 ± 0.001^Aa^	0.017 ± 0.001^Aa^
ALA (α-linolenic acid; C18:3)	18.17 ± 1.165^Aa^	11.57 ± 1.1400^Aa^	14.24 ± 0.7520^Aa^	13.19 ± 0.8085^Aa^
EPA (Eicosapentaenoic acid; C20:5)	0.158 ± 0.0172^Aa^	0.111 ± 0.0086^Aab^	0.109 ± 0.0067^Aab^	0.083 ± 0.0032^Ab^
DHA (Docosahexaenoic acid; C22:6)	0.460 ± 0.0421^Aa^	0.377 ± 0.0360^Aa^	0.352 ± 0.0206^Aa^	0.403 ± 0.0226^Aa^
Total ω-6	2.442 ± 0.1536^Aa^	1.572 ± 0.1501^Aa^	1.916 ± 0.1001^Aa^	1.773 ± 0.1080^Aa^
Total ω-3	18.78 ± 11.95 ^Aa^	12.05 ± 1.1738^Aa^	14.71 ± 0.7710^Aa^	13.68 ± 0.8284^Aa^
ω-6/ ω-3	0.137 ± 0.0021^Ab^	0.174 ± 0.0102^Aa^	0.130 ± 0.004^Ab^	0.006 ± 0.12^Ab^

*AD-Alzheimer disease

Results are reported in mean ± standard deviation

^ab^Different superscripts denote a statistically significant difference between controls of two different places and between Alzheimer patients of two places

^A^ superscripts denote a statistically significant difference between controls and Alzheimers patients of each place individually

### Correlation between dietary and plasma fatty acid concentrations

In Table 4, the correlation between plasma fatty acid and dietary fatty acid intake has been calculated. The concentration of AA and EPA was correlated with their respective dietary intakes (measured by repeated 24 h records). The dietary intakes of ALA, ω-3, and ω-6/ ω-3 were not correlated with their respective percentage in plasma lipids. No correlation was observed between the intake of 18 C precursors ALA and the plasma levels of their long chain derivatives (AA, EPA, and DHA).

**Table 4:** Correlation between consumption of food fatty acids with plasma fatty acid concentration

		Food							
Plasma		LA	AA	ALA	EPA	DHA	W6	W3	W6/W3
LA	Control	−0.332*	−.374*	0.084	− 0.378*	− 0.234	− 0.421**	− 0.030	− 0.102
	Alzheimer	0.041*	0.105	− 0.012	− 0.014	− 0.040	0.074	0.036	− 0.022
AA	Control	0.167	0.129	0.000	0.171	0.010	0.188	−0.022	0.144
	Alzheimer	−0.060	0.373*	−0.226	−0.127	− 0.027	−0.493**	− 0.230	−0.049
ALA	Control	−0.336	−0.200	0.227	−0.443	− 0.081	−0.304*	0.133	−0.291*
	Alzheimer	0.072	0.082	0.309	−0.024	0.198	−0.133	0.071	0.045
EPA	Control	0.024	0.019	0.110	0.315**	0.057	−0.040	0.090	−0.042
	Alzheimer	−0.033	−0.167	− 0.155	0.198*	0.018	−0.416*	− 0.132	0.017
DHA	Control	−0.369	−0.061	0.047	−0.069	0.322**	−0.198	− 0.077	−0.052
	Alzheimer	0.091	0.290*	0.024	0.027	0.228	0.162	0.106	−0.186
W6	Control	−0.32*	−0.208	0.232	−0.443**	0.035	0.350*	0.134	−0.325*
	Alzheimer	−0.188	−0.009	0.192	0.104	0.117	0.055*	0.215	−0.004
W3	Control	−0.339*	−0.201	0.227	−0.444**	− 0.076	−0.310**	0.131	−0.292*
	Alzheimer	−0.077	0.081	0.003	−0.028	0.209	−0.136	0.065	0.039
W6/W3	Control	0.090	0.012	−0.051	− 0.101	0.422**	− 0.008	0.104	− 0.048
	Alzheimer	−0.159	−0.090	− 0.042	0.024	0.036	0.215	−0.036	−0.222

*Correlations significant at *P* ≤ 0.05

**Correlations significant at *P* ≤ 0.001

## Discussions

In this study, the average age of women diagnosed with AD was more than 75 years, which was higher than the age reported in a previous study [20]. Age is the chief risk factor for AD [21, 22] whereas the most important inherited determinants known for this disease were the family history of AD and the presence of ApoE4 genotype [23]. For all age groups, the most comorbid diagnoses were diabetes, blood pressure, and cardiovascular diseases. Similar findings have been reported previously [24, 25]. Numerous chronic diseases, such as diabetes, hypertension, obesity, hyperlipidemia, and depression have been linked to AD [26, 27]. The applicability of each potential risk factor in moderating the age of onset or severity of AD development remains uncertain. It has been estimated that the threat of AD increases to between 50 and 65% in people with insulin resistance, particularly those with type 2 diabetes. Deficiency of insulin, besides impairing cognition, also seems to be involved in the formation of amyloid of plaque [28]. In the previous studies, various socioeconomic features [29], low education level [25, 30, 31], smoking [32], and alcohol [33] were identifiable risk factors that decrease the quality of life of patients [34]. In this study, the majority of women suffering from AD were either illiterate or just passed elementary levels. In previous studies, it has been noted that higher education level is related to lower rates of dementia and tended to protect against it [35, 36]. Body mass index (BMI) either lower than 21 or higher than 29 might increase risk of dementia or cognitive decline [37, 38], which might be because high BMI is associated with a generally higher cardiovascular risk and lower BMI reflected the start of the dementia processes and is associated with a greater cortical amyloid burden [39].

Proper lifestyle behaviors, including good nutrition and physical activity, are the foremost requirement in preventing chronic diseases and disabilities in old age [40, 41]. Cross-sectional studies provide evidence of the association between diet quality and prevalence of AD [42–44]. Dietary fat composition is an important factor in blood cholesterol profile and blood-brain barrier function and directs the significance of generating new dietary manipulation strategies to treat or prevent AD [45].

Malnourished AD patients or patients at risk of malnourishment were more impaired in basic and complex daily functioning than well-nourished AD patients, which suggests a relationship between nutritional status and basic and complex daily functioning in AD patients [46, 47]. Dietary parameters can be a potent tool for delaying the onset of AD or slowing its progression [45]. Various fatty acids, especially of exogenous origin (ω-3, ω-6, trans, and odd-numbered fatty acids), might provide the best quantitative estimates of their intakes, which can be measured in various blood fractions and tissues, such as plasma, adipose tissue, and erythrocytes [48].

It has been found that the percentage of subjects consuming fish daily was very low. Several epidemiological studies have reported that reduced levels of ω-3 fatty acids or reduced fish consumption are related to increased risk for age-related cognitive decline or dementia, such as AD [49]. The protective effect of fish might have several alternative explanations, such as a higher fish intake indicates a healthier dietary pattern or higher socioeconomic status, which in turn is linked with better cognitive performance [50]. Another possible explanation is that higher intake of fish might be related to lower intake of another type of fat, such as saturated fat, and that fish is a good source of various other nutrients that might also contribute to cognitive function improvement [51]. EPA and DHA are mainly sourced from marine fish [52]. Although EPA and DHA can also be synthesized from ALA, the conversion efficiency of ALA to EPA varies between 0.2 and 21%, and that of ALA to DHA varies between 0 and 9% [53, 54]. The concentration of plasma free fatty acids did not differ significantly across groups. In this study, it has been found that neither the concentration of free plasma DHA or ALA nor the concentration of omega 3 fatty acid was significantly lower in the AD group. Previously, it has been reported that DHA content in the brain is decreased in several neurodegenerative diseases [55], and significantly lower DHA levels were noticed in the blood plasma and brains of patients diagnosed with AD [56, 57]. Since DHA is a substrate of peroxisomal β-oxidation [58], it has been proposed that diminished plasma and tissue levels of DHA could be the consequence of peroxisomal dysfunction [55]. Reports from various epidemiological and observational studies show an inconsistent association between dietary intake of omega 3 fatty acids and the risk of dementia and AD. Some studies in humans have failed to find this association [59, 60], while other human studies indicate that increased intakes of omega 3 fatty acids from dietary sources are linked to a reduced risk of dementia and AD [61, 62]. In previous laboratory studies, animals fed diets enriched with n-3 PUFA had better regulation of neuronal membrane excitability [63, 64], greater fluidity of synaptic membranes [65], increased hippocampal nerve growth [66], and increased levels of neurotransmitters and higher density of neurotransmitter membrane receptors [67, 68], but the confirmation that plasma nutrient levels in AD are lower than in controls had thus far been lacking. This study shows that a large amount of EPA and DHA ingested came from food items other than fish, such as egg, meat, and oil, which is similar to the findings reported by Eissa et al. [69]. Samples were characterized by high linoleic acid intake and low α-linolenic acid intake, which is also consistent with the literature [70, 71].

ALA and LA are physiologically essential and complementary, but they compete as a substrate for the same enzyme-desaturase. The conversion rate of precursors to long-chain derivatives also depends on the levels of the dietary intake of the long chain PUFA [72, 73]. Conversion of ALA to longer chain fatty acid is reduced if the intake of ALA is too low. This is because it has to compete with the larger quantities of LA for the same enzyme. Since no correlation was detected between the dietary intakes of ALA and plasma levels of their respective bioconversion products, this suggests that the detected differences in the plasma levels of these long chains PUFA in our sample are determined by their habitual intake levels. This is probably due to the low conversion rate of linoleic and alpha-linolenic acids in humans [72]. Furthermore, the plasma levels of long-chain ω-3 PUFA was negatively correlated with that of linoleic. Blood or tissue PUFA levels are expected to differ with their intake levels because ω-6 or ω-3 PUFAs is not biosynthesized de novo. In previous studies, to assess the relationship between the plasma level of individual PUFA and their intakes, a significant correlation was observed between the dietary intakes of long-chain n-3 fatty acids EPA and DHA with their plasma levels [74, 75].

## Conclusion

The lower plasma level indicates lower impaired systemic availability of several nutrients. The finding shows that dietary intervention might play a role in the prevention of AD. Although no significant difference in the plasma level of fatty acids between two groups (control and AD) was observed, controlled subjects showed significantly higher intake of food rich in omega 3 fatty acids, which might support the fact that food and food supplement rich in omega 3 fatty acid (EPA and DHA) might delay the onset of AD. Education, income, overall health status, and the concentration of various fatty acids from food were higher and better in subjects SWH than NGH. This study is a small contribution to the search for an available and safe treatment for AD. However, further research is required to verify the role of n-3 fatty acids as a protective agent against cognitive decline.

## Abbreviations

AA: Arachidonic acid
AD: Alzheimer disease
ALA: α linolenic acid
BMI: Body mass index
DHA: Docosahexaenoic acid
EPA: Eicosapentaenoic acid
LA: Linoleic acid
MUFA: monounsaturated fatty acid
NGH: National Gaurd Health Affairs-King Abdulaziz Medical City, Riyadh
PUFA: Polyunsaturated fatty acid
SWH: Social Welfare Homes for Elderly
